# Senescence and Immunoregulation in the Tumor Microenvironment

**DOI:** 10.3389/fcell.2021.754069

**Published:** 2021-10-07

**Authors:** Megan K. Ruhland, Elise Alspach

**Affiliations:** ^1^Department of Cell, Developmental and Cancer Biology, Knight Cancer Institute, Oregon Health and Science University, Portland, OR, United States; ^2^Department of Molecular Microbiology and Immunology, Saint Louis University School of Medicine, St. Louis, MO, United States

**Keywords:** cancer immunology, senescence, stroma, aging, immune response

## Abstract

Immunotherapies have revolutionized cancer treatment, but despite the many lives that have been extended by these therapies many patients do not respond for reasons that are not well understood. The tumor microenvironment (TME) is comprised of heterogeneous cells that regulate tumor immune responses and likely influence immunotherapy response. Senescent (e.g., aged) stroma within the TME, and its expression of the senescence-associated secretory phenotype induces chronic inflammation that encourages tumor development and disease progression. Senescent environments also regulate the function of immune cells in ways that are decidedly protumorigenic. Here we discuss recent developments in senescence biology and the immunoregulatory functions of senescent stroma. Understanding the multitude of cell types present in the TME, including senescent stroma, will aid in the development of combinatorial therapeutic strategies to increase immunotherapy efficacy.

## Introduction

In the last 20 years there has been exponential growth in our understanding of the critical roles played by the immune system in regulating tumor development and mediating tumor rejection. This has culminated in revolutionary immunomodulating therapies that elicit robust and durable responses across diverse tumor types (Borghaei et al., 2015; Larkin et al., 2015; Motzer et al., 2015). While anti-tumor immunity is a concerted effort of both the innate and adaptive immune systems, much of the attention is focused on the activity of CD8^+^ T cells specific to tumor cell-expressed mutant peptides (neoantigens). Through cancer immunoediting, neoantigen-specific T cells prevent tumor development by killing tumor cell clones that express strong neoantigens, but also provide selective pressure for the outgrowth of the tumor cells that are not easily recognized by the immune system (Shankaran et al., 2001; DuPage et al., 2012; Matsushita et al., 2012). CD8^+^ T cells specific to neoantigens can mediate therapy-driven rejection in human patients (Wolfel et al., 1995; Robbins et al., 2013; Gubin et al., 2014; Strønen et al., 2016). Prognostic indicators like CD8^+^ T cell infiltration and cytotoxicity, and neoantigen burden have been used to predict response to immunotherapies targeting the PD-1 and CTLA-4 pathways but are imperfect (Rizvi et al., 2015; Van Allen et al., 2015; Spranger et al., 2016). Additionally, most patients still fail to see benefit following these therapies (Borghaei et al., 2015; Larkin et al., 2015; Motzer et al., 2015). One of the challenges faced in the field is to place the activity of immune cell subsets in the context of the broader TME to overcome immunotherapy resistance.

Rather than discrete aggregates of cancer cells, tumors are more akin to organs comprised of many heterogenous cell types (Anderson and Simon, 2020). Non-cancerous cells that make up tumors are collectively referred to as the TME, and include immune cell subsets (Binnewies et al., 2018), endothelial cells (Castermans and Griffioen, 2007; Goel et al., 2011), and stromal fibroblasts (Sahai et al., 2020). Cells within the TME directly interact with tumor cells, but also influence the function of other TME residents, creating a complex network of interactions that ultimately determines tumor fate. TME cell subsets can promote or impede tumor progression. For example, for every antitumorigenic immune cell subset there is a protumorigenic alter ego (Gajewski et al., 2013; Greten and Grivennikov, 2019). Tumor vasculature is critical for the delivery of therapeutics including immunotherapies, but also delivers nutrients to tumors and can function as a barrier for immune cell infiltration (Goel et al., 2011; Turley et al., 2015). While all cells of the TME play important roles in tumor progression and can influence anti-tumor immunity, the focus of this review is on tumor-associated stromal fibroblast populations.

Studies in the last decade have increased our understanding of cancer-associated fibroblasts (CAF). What was once a homogeneous tumor-promoting population of cells is now known to be comprised of distinct cellular subsets with both tumor-permissive and suppressive functions that can potently impact anti-tumor immunity (Özdemir et al., 2014; Rhim et al., 2014; Dominguez et al., 2020; Grauel et al., 2020; Kieffer et al., 2020). CAF are generally immunosuppressive and enhance the recruitment and pro-tumorigenic phenotypes of myeloid cells through expression of chemokines and cytokines including CXCL and CCL family members, IL-6, IL-10, and TGFβ (Monteran and Erez, 2019). CAF also suppress T cell function by promoting infiltration and polarization of regulatory CD4^+^ T cells, expression of immune checkpoint molecules like PD-L1, and aberrant antigen presentation (Nazareth et al., 2007; Costa et al., 2018; Lakins et al., 2018; Elyada et al., 2019). Importantly, the collective CAF phenotype is not the only fibroblast population found in the TME. Senescent fibroblasts are also incorporated into tumors (Alspach et al., 2013). Senescent fibroblasts are permanently growth arrested but metabolically active and share many similarities with CAF. However, the two phenotypes have distinct differences with important implications for therapeutic targeting. Detailed reviews regarding the status of CAF biology are available (Valkenburg et al., 2018; Gieniec et al., 2019; Sahai et al., 2020). Here we will discuss senescent stroma biology, recent findings that underlie its importance in driving disease and its potential for mediating immunosuppression within tumors.

## What is Senescence?

Following the advent of tissue culture techniques in the early 1900’s, the dogma surrounding the replicative lifespan of cells *in vitro* stated that they were immortal. However, in 1961 Leonard Hayflick and Paul Moorhead published a seminal report demonstrating that genetically normal human fibroblasts had a finite replicative lifespan *in vitro*, and cells entered a state of permanent growth arrest once this point was reached (Hayflick and Moorhead, 1961; Shay and Wright, 2000). These findings are now known as the “Hayflick limit”, and the state of permanent growth arrest that Hayflick observed is now called cellular senescence. Our understanding of what senescence entails has greatly evolved over the ensuing decades.

### Features of Senescent Cells

Senescent cells display gross phenotypic changes including an enlarged, flattened morphology, distinct stress fibers, and enlarged nuclei, as well as changes in lysosome function and altered transcriptional profiles (which will be discussed in more detail below) (Alspach et al., 2013; Hernandez-Segura et al., 2018). Senescent cells are arrested in G_1_–G_2_ of the cell cycle *via* the activation of regulatory pathways including p53/p21^WAF1/CIP1^, p16^INK4A^/pRB, and p27^KIP1^ (Atadja et al., 1995; Alcorta et al., 1996; Ruhland et al., 2016). Recently, the “permanent” nature of the senescence growth arrest has been called into question following findings that senescent tumor cells can reenter the cell cycle following inhibition of p53, or activation of H3K9me3 demethylases (Milanovic et al., 2018; Yu et al., 2018). Senescent cells are generally resistant to apoptosis, although apoptosis of senescent cells has recently been demonstrated using small molecule inhibitors of the anti-apoptotic BCL-2 protein family, and D-retro inverso (DRI) peptide mediated disruption of interactions between FOXO4 and p53 that result in the activation of caspase3/7 (Zhu et al., 2016; Baar et al., 2017). These findings have added additional layers of nuance to the definition of senescence to create a more dynamic and heterogenous picture of this cell state.

### Inducers of Cellular Senescence

In general, senescence is induced *via* persistent DNA damage signaling that activates cell cycle regulatory pathways (Campisi and d’Adda di Fagagna, 2007). Stress-induced premature senescence (SIPS) can be induced *in vitro* through ionizing radiation (Palacio et al., 2019), treatment with genotoxic drugs like bleomycin and doxorubicin (Alspach et al., 2014; Demaria et al., 2017), and reactive oxygen species (ROS)-producing chemicals like hydrogen peroxide (Kim et al., 2019). The replicative senescence originally observed by Hayflick is induced by progressive telomere attrition over many rounds of cell division that eventually results in irreparable DNA damage signaling from the ends of chromosomes (Bodnar et al., 1998; Fumagalli et al., 2012). Interestingly, telomeres are the primary genetic location of persistent DNA damage in SIPS driven by both general DNA damaging agents and replicative senescence (Hewitt et al., 2012). More recently, a genome-wide CRISPR screen identified the histone acetyltransferase KAT7 as a novel regulator of senescence induction (Wang et al., 2021).

Inducers of senescence *in vivo* follow the general theme of DNA damaging agents. Over the course of natural aging and telomere shortening, senescent cells accumulate in mouse, human, and non-human primate tissues (Dimri et al., 1995; Herbig et al., 2006; Baker et al., 2016). The activation of oncogenes, including Ras, Braf, and E2F1, and inactivation of tumor suppressors including PTEN, result in uncontrolled cell division. This creates genotoxic stress that can lead to persistent DNA damage and senescence induction (Di Micco et al., 2006; Mallette et al., 2007; Kumari and Jat, 2021). Systemic chemotherapy was recently shown to induce senescence in many tissues in mice including lung, skin, and liver, and within the stroma of human prostate tumors and head and neck squamous cell carcinomas (Mellone et al., 2016; Demaria et al., 2017; Xu et al., 2019). Type 1 inflammatory responses driven by Simian virus 40 large T antigen (Tag)-specific CD4^+^ T cells have also been shown to induce senescence of Tag-expressing tumor cells in an IFNγ and TNFα dependent manner (Braumüller et al., 2013), and it is reasonable to hypothesize that type 1 inflammation is capable of inducing senescence throughout the TME. Hypoxic conditions like those found in many tumors increase cellular ROS production and oxidative stress. It is possible that these conditions in the TME represent another way in which senescence is established in tumors, although this has not been formally demonstrated. The ways in which senescent stroma can be induced distinguishes these cells from CAF. CAF induction is predominantly dependent on signaling from tumor cells (Sahai et al., 2020), while senescence can be established through tumor-independent mechanisms and can impact many disease states, including all aspects of cancer initiation, development, and progression.

### Identifying Senescent Stroma

Elucidating the role of senescent stroma in tumor promotion and regulation of anti-tumor immunity is complicated by the challenge of identifying both the fibroblast cell type and the senescent state *in vivo*. Fibroblasts lack specific lineage markers and instead are defined by the absence of markers that define epithelial cells, endothelial cells, and leukocytes (e.g., EpCAM, CD31, and CD45, respectively) (Sahai et al., 2020). The absence of defining markers of other cell types is often combined with vimentin and/or α-smooth muscle actin, which are expressed by fibroblasts and other mesenchymal cells (Sahai et al., 2020).

One of the most common markers used to identify senescent cells is senescence-associated β-galactosidase (SA β-gal) staining, which preferentially labels senescent cells based on their altered lysosomal activity (Debacq-Chainiaux et al., 2009). Senescence can also be identified based on the increased expression of the cell cycle inhibitor p16 *via* immunohistochemistry or gene expression analysis (Baker et al., 2011; Demaria et al., 2017; Xu et al., 2019). As discussed previously, senescent cells express an altered transcriptional profile termed the senescence-associated secretory phenotype (SASP), which can be used as an additional marker of the senescent state and mediates the biologic impacts of senescent cells (Table 1). The SASP is comprised of a group of coordinately upregulated chemokines, cytokines, growth factors, and modifiers of the extracellular matrix (Bavik et al., 2006; Coppé et al., 2008; Pazolli et al., 2009). While the specific components of the SASP may differ based on the mode of senescence induction or anatomical location (Kumari and Jat, 2021), upregulation of IL-6 is often used as a surrogate marker for overall SASP expression within tissues (Demaria et al., 2017). The secretory profile of senescent cells is highly overlapping with that of CAFs, and similar regulatory mechanisms for these profiles have been observed (Alspach et al., 2014).

**TABLE 1:** The immunoregulatory capabilities of a selection of factors found in the SASP.

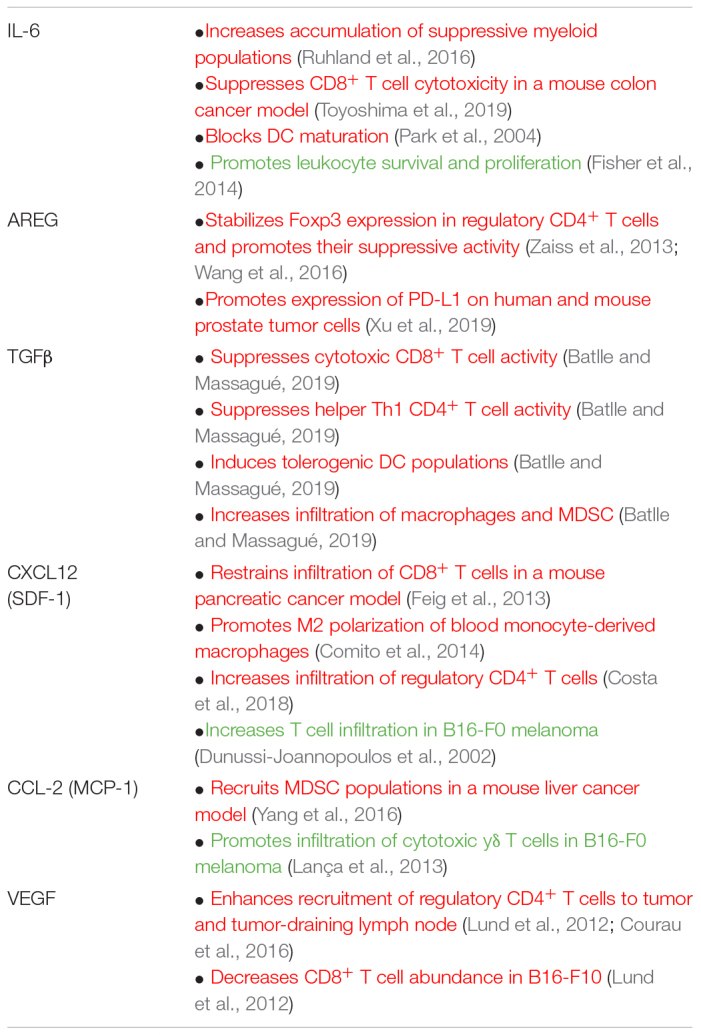

*While not exhaustive, this table shows examples of the many ways in which factors that can be upregulated upon senescence influence immune cell function in the tumor context. interleukin-6 (IL-6), amphiregulin (AREG), transforming growth factor beta (TGFβ), myeloid-derived suppressor cell (MDSC), C-X-C motif chemokine ligand 12 (CXCL-12), stromal cell derived factor 1 (SDF-1), C-C motif chemokine ligand 2 (CCL2), monocyte chemoattractant 1 (MCP-1), vascular endothelial growth factor 1 (VEGF). Red font indicates tumor promoting functions and green font indicates tumor suppressive functions.*

These traditional markers of fibroblasts and senescence can be used in combination to identify senescent cells in general and senescent tumor stroma, but they pose challenges for high dimensional profiling and next generation technologies. For example, the absence of specific surface markers makes identifying fibroblasts within the complex milieu of the TME difficult and SA β-gal staining cannot be used to identify senescent cells within single cell RNA sequencing datasets. While more amenable surface markers of senescence have recently been identified, including the urokinase-type plasminogen activator receptor (uPAR; Amor et al., 2020) and dipeptidyl peptidase 4 (DPP4/CD26) (Kim et al., 2017), a continuing challenge in the field is the development of a standardized panel of markers for the identification of senescent stroma.

## Senescence in Pathology

While cellular senescence is required for successful embryonic development (Muñoz-Espín et al., 2013; Domínguez-Bautista et al., 2021) and plays critical roles in wound healing (Demaria et al., 2014), most of what we know regarding the physiologic impacts of senescent cells is based in disease states. Because of early observations that senescence accumulates with age (Dimri et al., 1995), it was hypothesized that senescent cells contribute to the aging process. In the last decade, work by several labs has experimentally solidified the causal relationship between senescence and aging in genetic mouse models where senescent cells can be identified through reporter molecule expression and selectively depleted (Baker et al., 2011; Demaria et al., 2017). In INK-ATTAC mice the p16 promoter drives expression of a transgene encoding an FK506 binding protein (FKBP)-caspase 8 fusion protein that allows for conditional caspase 8 activation following treatment with the small molecule AP20187, and EGFP. Senescent cells in INK-ATTAC mice can thus be identified by EGFP expression and selectively depleted *via* caspase 8-mediated apoptosis (Baker et al., 2011). Expression of the INK-ATTAC transgene and selective depletion of senescent cells in either BubR1^H/H^ progeroid mice or naturally aged wild type mice result in (1) significantly delayed onset of age-related changes in spine curvature, eye function, and the composition of fat and muscle tissue (Baker et al., 2011); (2) enhanced renal and cardiac function (Baker et al., 2016); and (3) significantly prolonged lifespan (Baker et al., 2016). Recently, expression of the INK-ATTAC transgene in the MAPT^P301S^PS19 mouse model of neurodegenerative disease demonstrated accumulation of senescent cells in the brain, the clearance of which reduced neurofibrillary tangles and enhanced cognitive function (Bussian et al., 2018). The depletion of senescent cells in INK-ATTAC mice also resulted in significantly longer latency of spontaneously arising tumors (Baker et al., 2016). In the final sections of this review, we will discuss the complex relationship between tumorigenesis and senescence and the potential of senescent stroma to regulate anti-tumor immunity.

### Senescence and Tumors: A Paradoxical Relationship

One of the first biologic functions attributed to cellular senescence was tumor prevention. As discussed previously, oncogene activation and tumor suppressor inhibition are potent senescence inducers. While senescence is abundant in premalignant lesions, its loss upon progression to neoplastic disease is indicative of the requirement to overcome senescence for tumors to develop (Chen et al., 2005; Michaloglou et al., 2005; Bartkova et al., 2006). Generally, induction of senescence in incipient tumor cells prevents malignancy. Induction of senescence in the genetically normal host cells within the TME often has the opposite effect. Senescence of immune cells within the TME (immunosenescence) is only beginning to be understood but is generally thought to be tumor promoting particularly when it occurs within T cell populations (Montes et al., 2008; Ye et al., 2012). Detailed discussions of immunosenescence are available elsewhere (Aiello et al., 2019; Prieto and Baker, 2019), and we will restrict our focus to the protumorigenic impact of senescent stromal fibroblasts.

The interest in the potential of senescence to promote, rather than inhibit, tumor development was spurred by the fact that age is the greatest risk factor for the development of cancer. Work by Dr. Judith Campisi and others using co-transplantation of tumor cell lines with senescent or non-senescent fibroblast cell lines provided the foundational evidence of the tumor-promoting capability of stromal senescence. Implantation of preneoplastic skin, breast, and prostate cell lines of mouse and human origins with senescent fibroblasts results in more aggressive tumor outgrowth compared to the tumor development observed when these cells are implanted in the presence of non-senescent fibroblasts (Krtolica et al., 2001; Pazolli et al., 2009; Ruhland et al., 2016). Researchers more recently showed using the p16-3MR mouse model (which is similar to the INK-ATTAC model and allows for the selective depletion of senescent cells) that senescence can be established *via* systemic chemotherapy treatment in mice (treatment-induced senescence, TIS), and that this drives the recurrence of MMTV-PyMT breast cancer cells after surgical resection and enhances metastatic growth in the lungs (Demaria et al., 2017). Similarly, prostate cancer patients with higher levels of TIS within the tumor stroma experienced significantly shorter disease-free survival (Xu et al., 2019). Tumor promotion by senescent stroma is mediated predominantly by SASP factors, as the inhibition of SASP expression abrogates the ability of senescent fibroblasts to enhance tumor growth (Pazolli et al., 2009; Alspach et al., 2014). SASP factors drive chronic inflammation that predisposes tissues to tumor initiation, promote tumor cell proliferation and invasion, and condition metastatic sites (Parrinello et al., 2005; Tsai et al., 2005; Bavik et al., 2006; Coppé et al., 2006, 2008; Liu and Hornsby, 2007; Pazolli et al., 2009; Herranz et al., 2015; Demaria et al., 2017; Gonzalez-Meljem et al., 2018; Perkins et al., 2020).

However, it is important to note anti-tumorigenic activity of senescent stroma has been reported, particularly within liver tissue (Krizhanovsky et al., 2008; Lujambio et al., 2013). It is intriguing to hypothesize that, like the pro and anti-tumorigenic subpopulations of CAF recently identified, that senescent cells also exist as a gradient of subsets with opposing impacts on tumors. High dimensional analysis of senescent populations will need to be employed to determine the extent of heterogeneity within the senescent phenotype.

## Immunoregulatory Activities of Senescent Stroma

**FIGURE 1:**
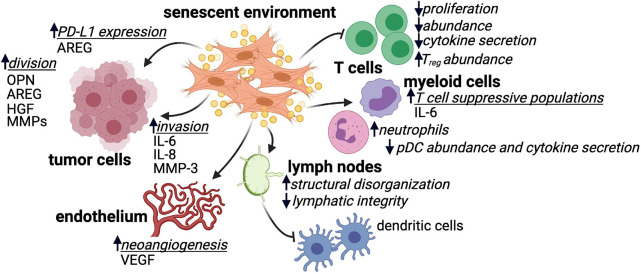
SASP factor secretion by senescent environments impacts many cell types. Senescent environments impact tumor cells, endothelial cells, various immune cell subsets, and lymphoid tissue in many ways that are or are hypothesized to be tumor promoting. Where identified, SASP factors produced by senescent environments that mediate these impacts are listed. Regulatory CD4^+^ T cell (Treg); plasmacytoid dendritic cell (pDC); amphiregulin (AREG); osteopontin (OPN); hepatocyte growth factor (HGF); matrix metalloproteinase (MMP); interleukin 6/8 (IL-6 and IL-8); vascular endothelial growth factor (VEGF).

In addition to direct interactions between senescent stroma and tumor cells, the SASP mediates crosstalk between senescent environments and a variety of immune cell populations (Figure 1). Many of these interactions indicate the potential of senescent stroma to suppress T cells, which are the main drivers of tumor rejection (Wolfel et al., 1995; Robbins et al., 2013; Gubin et al., 2014; Strønen et al., 2016; Alspach et al., 2019). Bone marrow transfers from young donor mice into naturally aged recipient animals resulted in CD4^+^ T cell populations that were significantly less proliferative and produced significantly less IL-2 upon stimulation *ex vivo* compared to parallel experiments using young recipients (Clise-Dwyer et al., 2007). Similar results were observed more recently using the p16-3MR mouse model where ionizing radiation resulted in senescence and SASP upregulation in splenocytes (Palacio et al., 2019). When senescent splenocytes were stimulated in allogeneic mixed lymphocyte reactions, defects in CD3^+^ T cell proliferation were observed that were dependent on secreted SASP factors from the senescent splenic environment rather than intrinsic defects in T cell function (Palacio et al., 2019). In a tamoxifen inducible model of stromal senescence driven by expression of p27^KIP1^, the presence of senescent mouse skin fibroblasts resulted in increased infiltration of CD45^+^ cells (Ruhland et al., 2016). While this increased immune infiltrate contained lower frequencies of CD3^+^ T cells in general, the frequency of immunosuppressive regulatory Foxp3^+^ CD4^+^ T cells was significantly enhanced (Ruhland et al., 2016).

Cells of the innate immune system are also impacted by senescent or aged environments. The presence of senescent cells in mouse skin tissue increased the frequency of CD11b^+^Ly6G^hi^ myeloid cells that suppressed CD8^+^ T cell proliferation and IFNγ production *in vitro* (Ruhland et al., 2016). Similar observations were made in a mouse model of prostate cancer, where loss of PTEN induced tumor senescence and the establishment of a TME enriched in CD8^+^ T cell-suppressive myeloid cells (Toso et al., 2014). The senescence-mediated accumulation of suppressive CD11b^+^Ly6G^hi^ myeloid cells in mouse skin was dependent on expression of the SASP factor IL-6 (Ruhland et al., 2016). In aged individuals, myeloid populations are significantly skewed including increases in monocyte-derived macrophages and neutrophils and, conversely, profound decreases in plasmacytoid dendritic cells (pDCs; Mogilenko et al., 2021). The myeloid cells present in aged environments display altered cytokine production and functional responsiveness (Hearps et al., 2012). For example, in response to influenza infection, pDCs from older individuals secrete less IFNα compared to younger individuals (Jing et al., 2009).

The architecture of the lymph node, the headquarters of immune response initiation in many settings including cancer, is altered with age. The characteristic compartmentalized structure of the lymph node is critical for the generation of effective immune responses (Li et al., 2020; Kapoor et al., 2021). The number of fibroblastic reticular cells (FRCs) decreases in the lymph node with age, and the structural meshwork and extracellular matrix proteins provided by these stromal cells shows marked disorganization (Becklund et al., 2016). Additionally, lymphatics become leaky and display a decreased ability to support lymph flow and may ultimately contribute to altered antigen transport and delayed immune responses (Zolla et al., 2015). While it has yet to be shown that age-related lymph node dysregulation is the direct result of FRC senescence, a study in the setting of organ transplantation demonstrated that senescent FRCs, which accumulate following transplantation, drive disruption of lymph node architecture, increased collagen I deposition and the establishment of a proinflammatory environment (Li et al., 2020). FRCs are integral to the organization of the lymph node, and it is reasonable to hypothesize that senescence within this stromal population may contribute to defects in T cell and dendritic cell migration within the lymph node as well as the germinal center dysfunction and blunted humoral response seen in older individuals (Wagner et al., 2018). Considering the heterogeneity of stromal cells in the lymph node and their demonstrated role in facilitating appropriate immune responses (Knoblich et al., 2018; Rodda et al., 2018; Kapoor et al., 2021), extensive work is needed to understand the direct contribution of senescent stromal populations in the lymph node to peripheral anti-tumor immune dysfunction.

Finally, SASP factors expressed by senescent cells upregulate cell surface immunosuppressive proteins. Following treatment with genotoxic compounds, oncogene expression or ionizing radiation, mouse and human fibroblasts upregulated the non-canonical MHC molecule Qa-I^b^ or HLA-E, respectively (Pereira et al., 2019). HLA-E/Qa-I^b^ expression significantly reduced the cytotoxic activity of both natural killer cells and CD8^+^ T cells. Senescent fibroblast-conditioned media was sufficient to upregulate HLA-E/Qa-I^b^ on the surface of non-senescent fibroblasts, and this was driven, in part, by the SASP factor IL-6 (Pereira et al., 2019). In human prostate cancer samples, expression of SASP factor amphiregulin (AREG) by senescent stroma was correlated with increased tumor expression of PD-L1 (Xu et al., 2019). Furthermore, AREG expression by a human prostate fibroblast cell line was sufficient to promote PD-L1 expression on PC3 prostate cancer cells (Xu et al., 2019). The PD-1/PD-L1 pathway is a major driver of immunosuppression within the tumor microenvironment, and AREG-mediated upregulation of PD-L1 on tumor cells may explain the significantly reduced progression free survival observed in prostate cancer samples with higher levels of stromal senescence (Xu et al., 2019). Importantly, many of these immunosuppressive impacts of senescent cells occurred in the absence of tumor cells, indicating that senescent stroma may prevent immunosurveillance during the earliest stages of tumor initiation. This is particularly intriguing given recent evidence that cancer immunoediting occurs less efficiently in older patients (Castro et al., 2020).

## Discussion

The immunoregulatory capacity of the TME is clear, as is the immunosuppressive potential of senescent and aged stroma. While many examples of the suppressive potential of senescent cells have been demonstrated in settings outside the tumor context (e.g., the skin and the spleen), it is reasonable to hypothesize that senescent stroma will mediate similar processes within the TME. Furthermore, the impact of secreted SASP factors on one cell type (e.g., SASP factor-mediated upregulation of alternative MHC molecules on senescent fibroblasts) has the potential to impose the same changes on nearby cells like tumor cells in the TME.

An argument can be made that, compared to CAF populations that are often proliferative, the non-proliferative nature of senescent cells may make them minor players in tumor regulation. However, injection of small numbers of senescent cells into young mice was sufficient to induce age-related phenotypic changes (Xu et al., 2017, 2018). Additionally, clearance of senescence induced *via* natural aging or systemic chemotherapy (where senescence is presumably induced sporadically within tissues) was effective in significantly improving pathologies in mouse models (Baker et al., 2016; Baar et al., 2017; Demaria et al., 2017). These results argue that even small amounts of senescence within tissues, including the TME, have the potential to significantly impact disease outcome and present rationale for the therapeutic targeting of senescent cells.

Therapeutic interventions targeting the tumor stroma, particularly CAF, are currently in development to improve anti-tumor immunity (Chakravarthy et al., 2018; Hanley and Thomas, 2021). However, the unique characteristics of senescent cells may make them resistant to therapies developed to target CAF. For example, therapies intended to deplete CAF subsets may be ineffective in depleting senescent stroma which exhibits heightened apoptotic resistance. Strategies for targeting senescence *in vivo* are also being developed. Small molecule inhibitors of the antiapoptotic BCL-2 and BCL-XL like venetoclax and navitoclax have been shown to induce apoptosis preferentially in senescent cells (Demaria et al., 2017; Xu et al., 2018; Kirkland and Tchkonia, 2020). Glutaminase-1 (GLS1) is required for senescent cell viability, and inhibition of GLS1 was recently shown to deplete senescent cells (Johmura et al., 2021). Senescence-targeting immunotherapy in the form of uPAR-specific chimeric antigen receptor (CAR) T cells was effective in clearing oncogene-induced senescence in mouse liver (Amor et al., 2020). The ability of senescence-targeting therapies to improve anti-tumor immunity should be determined. In this way, strategies aimed at depleting senescence can be added to our arsenal of stroma-targeting therapies to expand the benefits of immunotherapies to more patients.

Our understanding of senescence biology has advanced significantly since Hayflick determined his limit. However, while the impact of senescence on tumor growth is established (Alspach et al., 2013; Baker et al., 2016; Demaria et al., 2017; Xu et al., 2019), many outstanding questions remain. Because of the challenges surrounding its identification, the true burden of senescence within the TME, and whether senescence induction in the TME varies in different tumor contexts (e.g., inflamed versus non-inflamed, treatment responsive versus treatment resistant) is not clear. While senescence impacts many immune cell subsets, the mechanisms employed are unknown. A better understanding of the immunoregulatory role played by senescence will mean a better understanding of immunoregulation in the TME, and it is the entirety of the TME that determines tumor fate (Dunussi-Joannopoulos et al., 2002; Park et al., 2004; Parrinello et al., 2005; Tsai et al., 2005; Lund et al., 2012; Feig et al., 2013; Lança et al., 2013; Zaiss et al., 2013; Comito et al., 2014; Fisher et al., 2014; Herranz et al., 2015; Courau et al., 2016; Wang et al., 2016; Yang et al., 2016; Gonzalez-Meljem et al., 2018; Batlle and Massagué, 2019; Toyoshima et al., 2019).

## Author Contributions

EA conceived of the review. EA and MR wrote and edited the final manuscript. Both authors contributed to the article and approved the final submission.

## Conflict of Interest

The authors declare that the research was conducted in the absence of any commercial or financial relationships that could be construed as a potential conflict of interest.

## Publisher’s Note

All claims expressed in this article are solely those of the authors and do not necessarily represent those of their affiliated organizations, or those of the publisher, the editors and the reviewers. Any product that may be evaluated in this article, or claim that may be made by its manufacturer, is not guaranteed or endorsed by the publisher.
